# Cynophobia

**Published:** 1887-08

**Authors:** 


### *CYNOPHOBIA.*

An epidemic of disease hitherto undescribed in medical literature is now prevalent in Buffalo, raging with especial severity among the aldermen. The affection is characterized principally by a morbid fear of dogs, and hence we have denominated it *cynophobia.* We find absolutely nothing about the disease in writings of the ancients, though Virgil alludes to it in a vague manner when describing the evil effects of Sirius, the dogstar, upon mankind:

> "*Sirius ardor,*
> *Ille sitim morbosque ferens mortalibus aegris*
> *Nascitur, et laevo contristat lumine coelum.*"

It will be observed that it is the men that are afflicted with "the thirst and diseases" when dogstar appears, and not the dogs. In the "History of the Epidemics of the Middle Ages," no allusion to any malady resembling cynophobia was found. We may therefore safely assume that it is essentially a modern disease as an epidemic, although it has always been epizootic with cats, foxes and some other varieties of game. Aristotle tells us that "Dogs are subject to rabies; it makes them mad; all animals that they bite also become mad, with the exception of man." As the ancients were not afraid of acquiring hydrophobia they were not afraid of dogs, and consequently could not have had cynophobia.

The causes of the disease are somewhat obscure. As the affection is most prevalent when the dogstar holds sway in the heavens, we are tempted to seek an astronomical origin of the disease, and attribute it, as Virgil does other ills, to the malign influence of Sirius. The excessive use of alcoholic drinks in hot weather may also be mentioned as a cause; the fact that the aldermen are most seriously affected giving some color to this hypothesis. We are informed that some of our "City Fathers" are so troubled with alcoholic hallucinations that they can distinguish *in carne* dogs from the *in mente* variety only by striking at them with their canes. The *in carne* dog will bite while the *in mente* dog does not move. They accordingly fear all dogs.

Cynophobia is characterized by the following symptoms:

1. A delusion that dogs have hydrophobia, when it is a well-established fact that this symptom of rabies, fear of water, is never found in the canine species, only men and cats being thus affected.

2. A delusion that dogs are peculiarly liable to become rabid in hot weather, when there is no evidence that climate or temperature have anything to do with the development of the disease. Audry observed in 1780 that rabies was most prevalent in winter. The distinguished French veterinarian, Bouley, has tabulated 3,096 cases occurring in dogs, and finds them distributed through the seasons as follows:

| | Winter. | Spring. | Summer. | Autumn. |
|---|---|---|---|---|
| Cases, . . . . . . . | 755 | 857 | 788 | 696 |
| Per cent., . . . . . . | 24¼ | 27¾ | 25½ | 22½ |

The cases are quite evenly distributed throughout the year. The slight excess occurring in the spring is not due to any meteorological conditions.

> "In the spring the young man's fancy
> Lightly turns to thoughts of love."

It is the same with the dogs, and as female society is somewhat limited among them, their rivalry is so much the greater, their jealousies more bitter, and their fights more frequent. If any quarrelsome lover happens to be infected with the microbe of Pasteur, he will communicate it to more of his associates than he would at other seasons of the year. If it were possible for him to have mucous syphilides in his mouth, this affection would likewise be most prevalent in the spring.

3. A delusion that rabies will develop spontaneously in hot weather. This hypothesis has long been exploded. Dupuytren, Majendie, Breschet and others have cruelly destroyed dogs by starvation aud exposure to the broiling sun, and yet no symptoms resembling rabies were developed. It has been demonstrated that the disease is propagated by inoculation only. People affected with cynophobia believe that all dog-bites are followed by the development of hydrophobia unless active preventive measures are adopted. When a dog has rubbed his tooth against them, they hasten to the nearest doctor who gratifies their whim by burning off an inch or so of skin with caustic, when they depart with sore legs and contented hearts. If, however, they are rich in this world's goods (common sense excepted), they take a pleasant journey to Paris to be treated by Monsieur Pasteur, where they run great risk of acquiring the very disease they so much dread.

4. Lastly, cynophobia is characterized by a delusion that epidemics of hydrophobia may be effectually averted by employing dog-catchers to entrap a few stray curs in a city, and imprisoning them in a dog pound. Our city is now spending $150 a month for this purpose, owing to the epidemic of cynophobia, which has attacked the Common Council. Two dog-catchers and a pound-keeper are employed at a salary of $50 per month each. (District physician receives the munificent salary of $33 per month.)

No fatal cases of cynophobia have yet been reported, and as a consequence the pathology of the disease is still a mystery. The pound-keeper, however, was so severely affected that he imbibed large quantities of *spiritus frumenti* as a prophylactic measure against rabies, and it was found necessary to commit him to the penitentiary for thirty days in order that the disease might not have a fatal issue.

Any further developments regarding this new epidemic will be furnished our readers.

